# Miscellany

**Published:** 1850-03

**Authors:** 


### ARTICLE V.

### MISCELLANY.

After a suspension that has continued since the great fire in St. Louis, last spring, by which its publication office was destroyed, the St. Louis Medical and Surgical Journal is again issued. The only change we observe it to have undergone, is the insertion of the name of our friend, Prof. Jno. B. Johnson, as co-Editor, in the place of Prof. McDowell. We heartily welcome it back to our list of exchanges.

*Rush Medical College.*—At the recent commencement of this Institution held Feb. 7th, the Degree of Doctor of Medicine was conferred upon Forty-Four gentlemen, a list of whose names will appear in our next.

The two medical schools of St. Louis had each 110 students during the past session, making 220 in both, which is a larger number than heretofore. The advantages of St. Louis for giving medical instruction are beginning to be appreciated.

The editor of the Western Lancet thinks the ticket fees in the Medical College of Ohio, in which he is a Professor, might with propriety be reduced a little. We think so too; nor would it derogate in the least from the high reputation of the school for giving sound and thorough instruction.

S. Hanbury Smith, M.D., of Cincinnati, Ohio, has succeeded the late lamented Butterfield, both in the Chair of Practice in the Starling Medical College, and as Editor of the Ohio Medical and Surgical Journal.

*American Medical Association.*—Our friends will observe in a preceeding article, a notice of the Committee of arrangements of this Association, that gives them the conditions of membership, &c., &c.

We hope for the credit of the West, and the good of the profession in it, that all her Medical Societies and other Institutions entitled to representation, will send delegates to this great National Medical and Scientific festival.

The National Convention for revising the Pharmacopœia of of the United States, will commence its sittings at the city of Washington on the first day of May next. We hope that the importance of this Convention will not be overlooked. The various improvements in Chemistry and Pharmacy render it necessary that the Pharmacopœia should be so modified as to form a true exponent of the present condition of Pharmaceutical Science.

